# The pains amidst starting a new life: investigating adjustment disorder in Hong Kong migrants’ transition to the UK

**DOI:** 10.1038/s41598-025-24311-1

**Published:** 2025-11-18

**Authors:** Ricci Yue, Bobo H. P. Lau

**Affiliations:** 1 Institute for Sustainable Resources, University College London, WC1H ONN London, UK; 2 Department of Counselling and Psychology, Hong Kong Shue Yan University, North Point, Hong Kong

**Keywords:** Psychology, Human behaviour

## Abstract

Migration can be a stressful process. While conventional studies often focus on voluntary migration, reluctant migration and its association with adjustment disorder remain underexplored. This study examined the prevalence rate and determinants of adjustment disorder among Hong Kong migrants arriving in the UK after mid-2020, following the UK’s announcement of a humanitarian visa route for eligible Hong Kong citizens, namely the British National (Overseas) Visa Scheme. An online survey was conducted on 1,310 valid Hong Kong migrants. Findings indicate 31.8% of the respondents were likely experiencing adjustment disorder and the disorder remains highly prevalent 2 years after their arrival. The most common life stressors included moving to a new home (41.1%), language barrier (32.9%), pressure from cultural differences (29.7%), financial problems (27.6%), and career break due to migration (24.8%). Chronic stressors are statistically significant in screening for adjustment disorder. Multivariate logistic regression models suggested Hong Kong migrants with lower education backgrounds and pre-existing mental illness are more likely to suffer from adjustment disorder. This study enriches extant understanding on the mental health toll behind reluctant migration through the perspective of adjustment disorder. We highlighted potential policy gaps for migrants arriving through humanitarian visas.

**Supplementary Information:**

The online version contains supplementary material available at 10.1038/s41598-025-24311-1.

## Introduction

The target of Sustainable Development Goal 3 (SDG3) aims to ensure healthy lives and promote well-being for all at all ages by 2030, yet recent crises like the pandemic and escalating global conflicts present significant obstacles, particularly for migrants. The rising number of conflicts and human rights violations have led to unprecedented levels of forced displacement - the highest since World War II - with trends suggesting further escalation^1^. Existing research on migrants’ well-being often observed that immigrants may present better mental health than native-born citizens, which is usually labelled as the ‘healthy migrant effect’^2^. For instance, Wu and Schimmele^3^, in their examination of the depression rates among migrants in Canada, found that visible minority immigrants were healthier, despite a gradual increase in the depression rate for several decades soon after their arrival. Escobar et al.^4^ concluded a similar trend among US Hispanic populations, despite the substantial socioeconomic challenges faced by Mexican-born migrants. This initial mental health advantage is sometimes linked to the characteristics of those who choose to migrate, such as being mentally more resilient than others^3^.

However, recent studies have begun to challenge whether this initial observation holds true for all migrant groups, particularly those who are forced or reluctant to migrate, raising doubts about the consistency of this pattern^5,6^. Forced and reluctant migrants may not exhibit the positive initial mental health profile; instead, they can be susceptible to mental health crises despite their departure from their home countries^7^. For reluctant migrants, the potential uncertainty associated with restarting a new life in an unfamiliar environment under abrupt circumstances can result in daunting distress. Established social sciences framework such as Berry’s acculturation model^8^ and Lazarus and Folkman’s model^9^ for stress and coping provide the foundational lens of human adaptation to migration under stress and crisis. The former model posits that acculturation is a dynamic process, influenced by a myriad of personal and social factors, between retaining own heritage culture and adopting to the new culture, with integration, meaning being able to maintain own heritage culture while participating in the host culture, being a favourable outcome. The latter model highlights the subjective nature of stressors in the process of acculturation and the experience of stress as perceived lack of resource for handling the taxing situations. Empirical studies have also examined migrant stress and anxiety resulting from racial discrimination^10^, family separation^11^, cultural bereavement^12^ and the process of migration itself^13^. Post-displacement stress, coupled with experienced trauma, has been shown to be robustly associated with an elevated risk of mental disorders^14^. To exacerbate their struggles, migrants often face a heightened vulnerability compared to other populations in terms of access to health services in their host countries^15^.

In the context of the United Kingdom (UK), a recent report from the Mental Health Foundation highlights that both pre-migration experiences (e.g., torture, war, imprisonment, assaults) and post-migration stressors (e.g., financial insecurity, unemployment, inadequate housing, social isolation, prejudice, etc.) combined to jeopardize migrants’ mental health, putting asylum seekers and refugees in heightened vulnerability^16^. This is echoing older studies in the UK – Turner et al.^17^ reported up to 32.1% of their Kosovan Albanian migrants scored positive for PTSD occurrence, and Bogic et al.^18^ found that 56.6% of their refugee sample were presented with a mental disorder. However, as Burns et al.^19^ reviewed, most studies on migrants in the UK have focused on infectious diseases and general mental health outcomes. While there has been a special emphasis on accessibility to different health services, there lacks a nuanced view over how different groups of migrants (e.g., from different origins, asylum seekers versus refugees versus migrants from other visas schemes, migrant workers) fare differently.

## Adjustment disorder among migrants

Migrants’ mental health has often been evaluated by the presence of depressive, anxiety, post-traumatic stress, and psychotic disorders^20,21^. Yet, little is understood from the perspective of adjustment disorder (AD), despite research indicating its high prevalence among individuals experiencing life-changing events^22^. AD is characterized by a repertoire of affective, behavioural, and cognitive reactions which manifest maladaptive stress responses that emerged within the three months of the appearance of (an) identifiable stressor(s)^23–25^. Unlike post-traumatic stress disorder (PTSD), the stressors involved in AD are often non-life-threatening but personally salient, such as loss of relationships (e.g., divorce, family conflicts), work (e.g., unemployment, change in occupational setting) or health (e.g., onset of chronic illness, injury, physical disability). AD has an estimated prevalence of about 1% among the general public^24^, but elevates to much higher rates in populations facing an identifiable stressor (e.g., 27% in recent unemployment^25^; 18% in bereavement^26^;), or in consultation-liaison settings (e.g., 15–19% in oncological care, 32% in emergency department; 12% in psychiatric consultations)^27,28^. The systematic review by Morgan et al.^29^ found that female gender, younger age, unemployment, stress, physical illness, the lack of social support, and pre-existing mental disorders elevate the risk of AD.

AD ranked 7th among psychiatrists^30^ and 9th in clinical psychologists^31^ on its perceived frequencies of appearance in clinical settings, which are above borderline personality disorders, PTSD, and non-organic sleep disorders. Yet, it has relatively low ratings in terms of its ease of use and goodness of fit in clinical practice and is often treated as a ‘residual diagnosis’ considered only when the severity of the symptoms fails to reach the diagnostic criteria of other mental disorders such as major depressive disorder or anxiety disorders. Recently, the diagnosis of AD is made clearer with the introduction of the 11th revision of the International Classification of Diseases (ICD-11) criterion that elucidates more positive symptoms for clinicians to look for, including preoccupation of the stressor, exhibited as excessive worry, recurrent distressing thoughts and constant rumination, as well as failure to adapt, indicated by significant functional impairments and disturbances. This may heighten clinicians’ awareness toward AD diagnosis, especially considering the diagnosis’s strong predictive validity toward suicidality^32^ and exacerbation or relapse of pre-existing mental disorders^33^.

At present, seldom has the diagnosis been used for capturing the stress experienced by migrants, which is often exemplified in a complex combination of personally salient relationship-, occupational-, health- and acculturation-related stressors. An exception would be a study by Zaiontz et al.^34^ which involved clinical interviews with a group of native English-speaking immigrants in Milan who sought mental health support. In their two consecutive cohorts of clients seeking consultations in 2008 and 2009, AD reached 41–56%, while more than half of the clients with AD possess a history of mental disorder, and that comorbidity reaches 85% for these clients. Although the American Psychiatric Association specified AD to dissipate within six months of the onset of the disturbances, Morgan et al.^29^ found baseline AD diagnosis to be robustly related to the emergence of a mental disorder in the next 9 months to 10 years, indicating a much lengthier illness course. AD could be a gateway to more severe mental disorders, such as PTSD and major depressive disorder^33,35^.

### The current study

Although AD is often treated as a subthreshold or less severe diagnosis, the clinical reality supports the need for evaluating the diagnosis as it may effectively signpost increased risk of relapse of pre-existing mental illness comorbidity, and therefore utilization of mental health support, especially among vulnerable populations such as migrants who often have difficulty accessing these services. The current study presents the condition of migrants from Hong Kong, which has a distinctive socio-cultural-economic profile from samples of previous British migrant studies^19^. First, this group of migrants had some exposure to the British culture, because Hong Kong was under the British colonial rule before 1997. Second, most migrants from Hong Kong have basic proficiency in English, as the language is a compulsory subject in the education system in Hong Kong, leading to potentially less language barrier than migrants from more culturally-distanced backgrounds. However, like other migration waves, this wave of migration from Hong Kong to the UK was (partly) motivated by a recent political turmoil in 2019–2020. Due to the months-long social unrest and the COVID pandemic, the mental health of Hong Kong citizens has significantly deteriorated since 2019, as evidenced by the drastic increase in the rates of anxiety, depression, and post-traumatic stress symptoms and related disorders^36^, regardless of their political affiliations^37^. Beginning in January 2021, the UK government launched the Hong Kong British National (Overseas) BN(O) Visa Scheme following the enactment of Hong Kong’s National Security Laws (NSL) that criminalizes activities related to secession, subversion, terrorism, and collusion with foreign forces^32^. Many families have then emigrated from Hong Kong to the UK through this visa scheme. The impact assessment conducted by the Home Office^38^ estimated between 257,000 and 322,000 applications within the first five years of the visa scheme. As of December 2024, the UK has issued over 200,000 BN(O) visas since 2021^39^. While it is natural for migrants to believe migrating to a new place would improve their mental well-being, multiple studies^40,41^ indicated an opposite reality especially with the implementation of the NSL in Hong Kong. In this light, this study utilized the data collected from an online survey in 2022 to assess the psychological adjustment among the Hong Kong migrant community arriving in the UK since the introduction of the British National (Overseas) (BN(O)) status. Specifically, we identified the prevalence and factors of AD for elaborating the psychological resilience of this ethnically homogenous but sizable migrant population.

## Methods

### Data

The data for this study primarily comes from a cross-sectional online survey conducted from 1 November 2022 to 15 December 2022. Eligible participants were Hong Kong migrants aged 18 or above who arrived in the UK through the BN(O) visa routes or other visa schemes after the announcement of BN(O) visa scheme in mid-2020. Since there was no established formal channel to reach out to the Hong Kong diaspora, participants were recruited through convenience sampling online. The advertisement, including the survey link, was distributed via diverse channels such as social media, church networks, and migrant-targeted support services. Potential participants were invited to take part in a study related to how Hong Kong migrants adjust to life in the UK. No incentives were provided. A screening question was introduced in the beginning of the survey to remove respondents who have not arrived the UK or who were residing outside the UK (Table S2). The survey was available in English and traditional Chinese and hosted on JISC Online Surveys. Informed consent was obtained prior to the start of the survey and for the publication of the results in an online open access format. Ethical approval was obtained from the University of Liverpool (Reference: 11313). All methods were performed in accordance with the Declaration of Helsinki. A total of 1,310 responses were received, which were then cleaned and prepared for analysis. Incomplete responses were excluded. At the time of data collection, approximately 130,000 BN(O) visa applicants had arrived in the UK^42^, indicating that the survey successfully captured responses from 1% of the BN(O) population.

### Variables

#### Dependent variable: adjustment disorder

The ADNM-8 was employed to assess the presence of AD. It is a theory-driven diagnostic algorithm designed to measure core AD symptoms, including preoccupation and failure to adapt. The questions aligned with the WHO Working Group for Disorders Associated with Stress proposals for ICD-11 AD diagnosis and were initially validated in 2013^43^. The ADNM-8 comprises two parts – a stressor list and a symptom list. The stressor list includes 19 life events capturing various potential stressors that were the most aggravating in the last 6 months. For this study, additional stressors including ‘racism,’ ‘language efficiency,’ ‘pressure of cultural difference,’ ‘career break due to migration,’ and ‘prospect of children’s education’ were added to fit the context of migration. The ‘criminal act’ stressor was modified to ‘risk of criminal prosecution’ to safeguard the privacy of the participants. These adjustments were informed by the key challenges faced by the Hong Kong diaspora in numerous studies^40,44,45^ and systematic reviews of stressors faced by migrants and refugees^46^. The symptom list comprises eight items measuring AD core symptoms on a 4-point Likert scale, with scale scores calculated by summing the corresponding items. A potential AD diagnosis is indicated by a combination of an item rated ≥ 3 and two items rated ≥ 2 in both core symptom subscales, along with a rating ≥ 3 on the impairment criterion (item 8). The Cronbach Alphas for the subscales were 0.93 (preoccupation) and 0.91 (failure to adapt), indicating high reliability within the sample. The average inter-item correlations for preoccupation and failure to adapt were 0.77 and 0.70 respectively, implying a high level of associations among the respective items.

#### Independent variables

The present study encompassed seven demographic variables (Table 1) commonly utilized in studies employing the ADNM-8^29^ or on migrants’ mental health^47^. Additionally, three questions were incorporated to assess the migration experience of Hong Kong migrants, including whether the participants had prior living experience in the UK, duration of residence in the UK, and their self-perceived level of integration. We included the code set used for each variable in Table S1 and the questionnaire in Table S2 in the appendix.

### Statistical analysis

The main analysis is structured into three sections. The first part presents the basic descriptive statistics for the symptom lists of AD, alongside the migration experience and demographic variables. To investigate whether AD symptoms varied among the sample residing in the UK for different durations, linear regression was conducted to assess the impact of time on symptoms. Additionally, the results of the theory-driven diagnostic algorithm for AD are presented.

The second part of the analysis delves into the stressor list. A binary logistic regression model was employed to scrutinize the stressors’ contribution to a probable AD diagnosis. Given the potential temporal fluctuation of the stressors, linear regression tests were utilized to discern the temporal patterns for each stressor.

The final segment of the analysis comprises three multivariate logistic regression models. The initial model (Model 1) assessed how demographic variables affect the odds of a probable AD diagnosis. The subsequent model (Model 2) introduced migration experience as independent variables to the first model. Model 3 integrated the predictors of Models 1 and 2 and evaluate the combined effects from demographic variables and migration experiences.

Parameter estimates for the second and third parts are converted into odds ratios for clarity, accompanied by 95% confidence intervals and significance levels. All statistical analyses are conducted using Stata v.16.

## Results

### Demography of respondents

The total sample made up of 1,310 respondents (See Table 1 for sample characteristics). The majority of the respondents were middle-aged adults between the ages of 40–44 (18.4%), 45–49 (19.4%) or 50–54 (12.5%). There were more female respondents (57.1%) than males (42.9%). The sample was highly educated, with almost 80% of them holding an undergraduate degree or above. 58.6% and 81.4% of them migrated with their children or family members, respectively. 18.5% and 11.7% of the sample had a chronic illness or had been troubled by a mental disorder in the past two years, respectively. Regarding their migration experience, 85% of the respondents had not lived in the UK before. The majority of respondents (92.3%) entered the UK with a BN(O) visa. The survey revealed that 31.6% of the respondents moved to the UK less than 6 months ago at the time of data collection. The majority of the sample had a positive perception of their level of integration (86.2%).

**Table 1:** Socio-demographic variables related to the Hong Kong migrants in this study.

Demographic attribute					
Age	*N*	%	Education	*N*	%
18–24	58	4.6	Junior secondary or lower	128	10.1
25–29	113	8.9	Higher secondary education	131	10.3
30–34	113	8.9	Undergraduate	629	49.5
34–39	148	11.6	Master or above	382	30.1
40–44	234	18.4			
45–49	247	19.4	Family with children		
50–54	159	12.5	Yes	785	58.6
55–59	104	8.2	No	555	41.4
Gender			Migrating with family member		
Male	537	42.9	Yes	1022	81.4
Female	711	57.1	No	234	18.6
Chronic illness record			Mental illness record		
Yes	232	18.5	Yes	147	11.7
No	1019	81.5	No	1110	88.3

Migration attribute					
Previous UK living experience	N	%	Type of visa entering UK	N	%
Yes	192	15.0	BN(O) visa	1189	92.3
No	1092	85.0	British citizen or spouse visa	48	3.7
			Work/global talent/student visa	25	1.9
Perceived level of integration			Others	26	2.1
Very poor	22	1.7			
Fair	58	4.5	Duration of residence		
Below average	97	7.5	0–6 months	480	31.6
Average	403	31.3	6–12 months	367	24.2
Above average	328	25.5	12–18 months	418	27.5
Good	317	24.6	18 months or more	253	16.7
Excellent	62	4.8			

### Prevalence and development of adjustment disorder over time

**Fig. 1:**
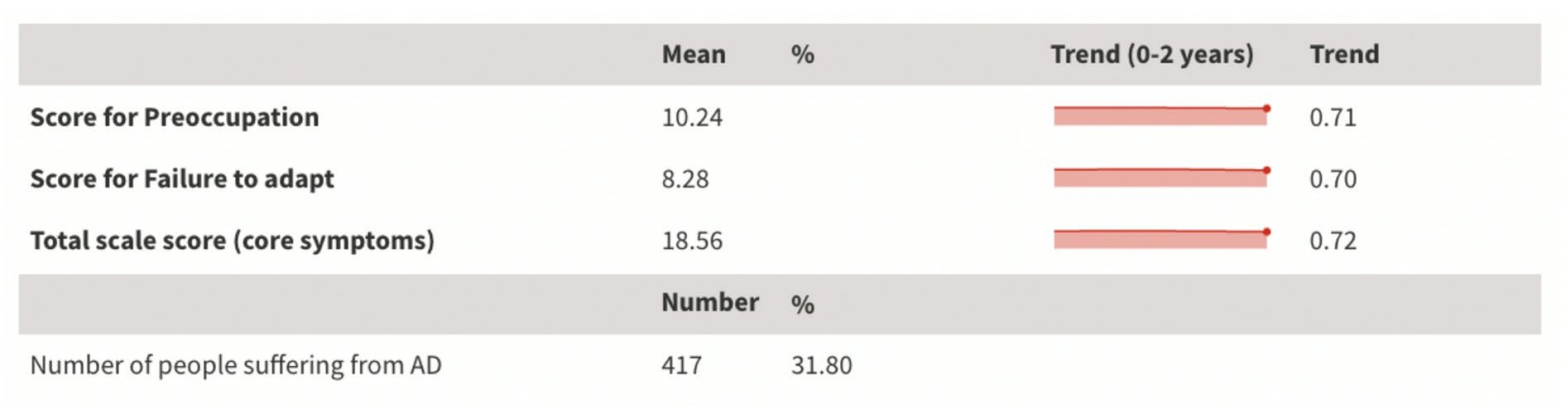
Results of ADNM-8 for the Hong Kong migrants. The life course of symptoms during the first two years of migration is visualized in pink.

AD was assessed by Adjustment Disorder – New Module 8 (ADNM-8) which is a theory-driven diagnostic algorithm designed to measure core AD symptoms, including preoccupation and failure to adapt. Figure 1 presents the descriptive statistics for the symptom list of AD. The sample scored an average of 10.24 and 8.32 in the preoccupation and the failure to adapt subscales respectively. The total scale score for ADNM-8 was 18.56.

To establish the prevalence of a probable AD diagnosis, the endorsement rates of core symptoms are reported further in Figure S1. A potential AD diagnosis is indicated by an item rated ≥ 3 and two items rated ≥ 2 in both core symptom subscales, combined with a rating ≥ 3 on the impairment criterion (item 8). Of all respondents, 74.1% and 47.4% rated at least one item ≥ 3 in the preoccupations subscale and failure to adapt subscale respectively. Based on this theory-driven diagnostic algorithm, 31.8% of the Hong Kong migrants were suffering from AD. Figure S1 also provides the mean score and score distribution of individual item under each subscale. Linear regression analysis showed no significant correlation between the duration of residence and the score for the two subscales, or the total scale score.

### Stressors after migration

**Fig. 2:**
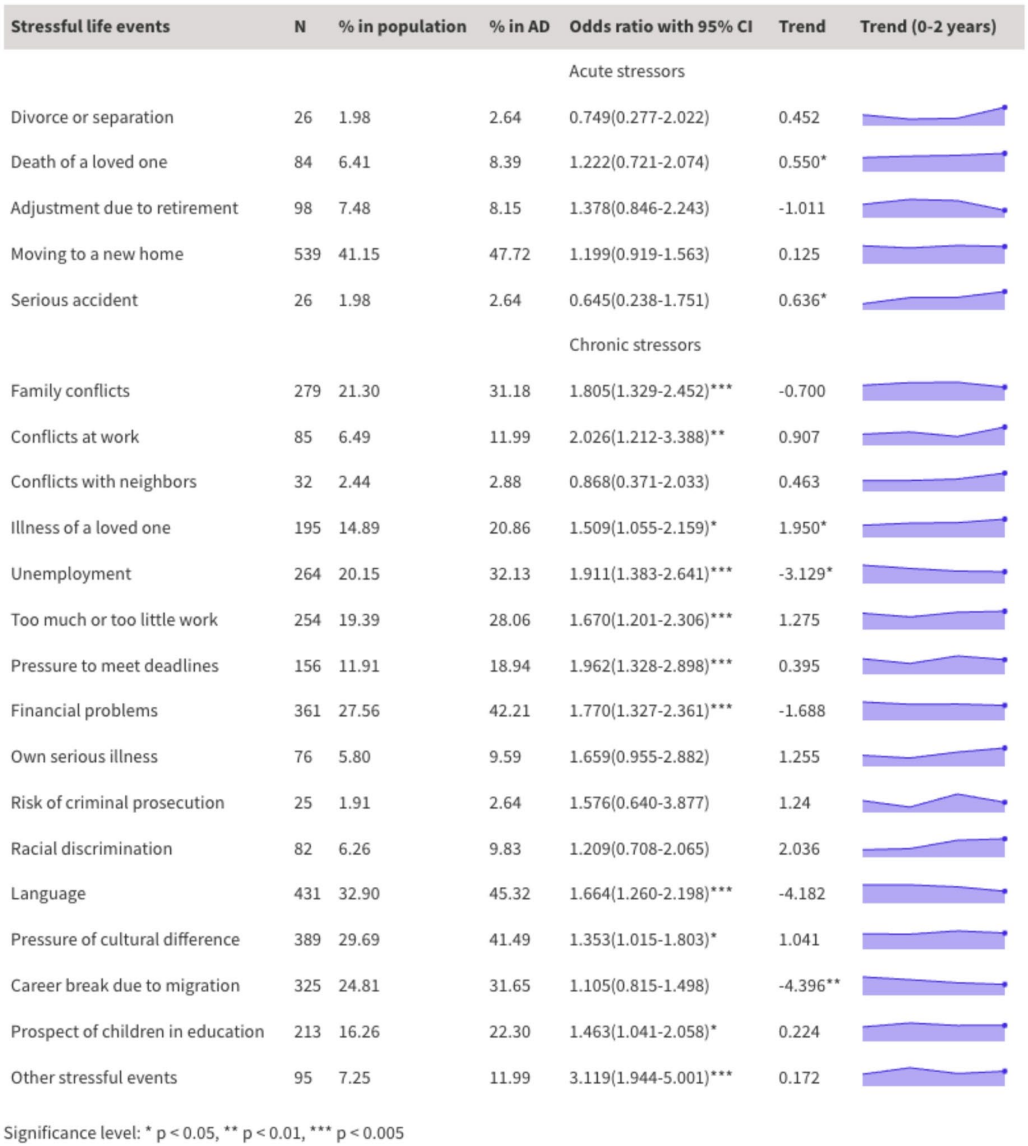
List of stressors in migration categorized as acute and chronic stressors. The stressors refers to the experience of respondents in their last six months. % in population indicates the proportion of respondents suffering from the stressor. % in AD indicates the proportion of respondents with adjustment disorder suffering from the stressor. Trend represents the results of linear regression analyses indicating significant trends of stressors over time. The life course of stressors during the first two years of migration is visualized in purple.

The most common life stressors in migration included moving to a new home (41.1%), language barrier (32.9%), pressure from cultural differences (29.7%), financial problems (27.6%) and career break due to migration (24.8%) (Fig. 2). For the population suffering from AD, the top stressful events include moving to a new home (47.7%), language barrier (45.3%), financial problems (42.2%), pressure of cultural difference (41.5%), and unemployment (32.1%). The majority of the sample (92.9%) reported at least one stressor. We conducted a series of linear regression analyses (Fig. 2) to determine whether the stressors exhibit change over time during migration. The results show that the likelihood of respondents experiencing significant stress due to the death of a loved one (Coeff: 0.550, *p* < 0.05), serious accident (Coeff: 0.645, *p* < 0.05), and illness of a loved one (Coeff: 1,950, *p* < 0.05) increased as time goes by. However, the probability of being affected by unemployment (Coeff: −3.129, *p* < 0.05) and career break due to migration (Coeff: −4.396, *p* < 0.01) significantly decreased in the first two years of relocation.

A binary logistic model was used to delineate the contribution of each stressor onto a probable diagnosis of AD. Family conflict (OR: 1.805, *p* < 0.005), conflicts at work (OR: 2.026, *p* < 0.01), illness of a loved one (OR: 1.509, *p* < 0.05), unemployment (OR: 1.911, *p* < 0.005), too much or too little work (OR: 1.670, *p* < 0.005), pressure to meet deadlines (OR: 1.962, *p* < 0.005), financial problems (OR: 1.770, *p* < 0.005), language barrier (OR: 1.664, *p* < 0.005), pressure of cultural difference (OR: 1.353, *p* < 0.05), prospect of children’s education (OR: 1.463, *p* < 0.05) and other stressful events (OR: 3.119, *p* < 0.005) are significant predictors of a probable AD diagnosis. The categorization of acute stressors and chronic stressors based on the recommendation of Kazlauskas et al.^48^ indicates that only chronic stressors predicted a probable AD diagnosis.

### Social determinants of adjustment disorder

Table 2 presents the results of three multivariate logistic regression models (Models 1 to 3). The first model estimated the predictive power of demographic variables on a potential AD diagnosis. Both lower education level (OR: 0.783, *p* < 0.005) and a pre-existing mental disorder (OR: 4.832, *p* < 0.005) were correlated with increased vulnerability to a probable AD diagnosis. In the second model (Model 2), migration-related variables were tested. Higher self-perceived level of integration (OR: 0.778, *p* < 0.005) was related to a lower likelihood of a probable AD diagnosis. Model 3 combined independent variables from both Model 1 and Model 2. Education level, pre-existing mental disorder and self-perceived level of integration remained statistically significant predictors of AD. Age (OR: 0.934, *p* < 0.05) emerged as significant, indicating that younger individuals were more vulnerable to a probable AD diagnosis.

**Table 2:** Results of multivariate logistic regression models on the social determinants of adjustment disorder. Significance level: * *p* < 0.05; ** *p* < 0.01;*** *p* < 0.001.

	Model 1 (demography) OR (95% CI)	Model 2 (migration attribute) OR (95% CI)	Model 3 (demography + migration attribute) OR (95% CI)
Age	0.942 (0.881–1.007)		0.934 (0.872–0.999)*
Gender	1.002 (0.769–1.306)		0.995 (9.761–1.301)
Education	0.783 (0.678–0.905)***		0.813 (0.702–0.941)**
Family with children	1.185 (0.866–1.619)		1.232 (0.898–1.692)
Migrating with family member	1.023 (0.718–1.456)		1.016 (0.711–1.452)
Chronic illness record	1.312 (0.930–1.851)		1.343 (0.949–1.901)
Mental illness record	4.832 (3.280–7.116)***		4.447 (3.006–6.581)***
Previous UK living experience		1.069 (0.739–1.546)	0.959 (0.657–1.400.657.400)
Duration of residence		1.049 (0.979–1.123)	1.007 (0.894–1.144)
Perceived level of integration		0.778 (0.705–0.859)***	0.806 (0.726–0.895)***
N	1146	1185	1142
LR chi-square	87.59	37.32	103.55
Pseudo R^2^	0.0613	0.0283	0.0729

## Discussion

### Adjustment disorder in the context of migration

Using data collected from a nationwide online survey conducted in late 2022, this study investigated the prevalence and factors of AD among Hong Kong migrants recently arriving in the UK. Our sample showed 31.8% of respondents could be suffering from AD, far exceeding the < 1% to 2% in general population^49,50^. This ratio also surpasses that of other high-risk populations, including recently laid-off individuals (27.3%)^25^, bereaved individuals (17.8%)^26^, quarantined populations during the early phase of COVID (14%)^51^, or patients in palliative-care settings (15.4%)^52^. Figure 1 further indicated a notably high rate of preoccupation and a moderately high rate of failure to adapt. This suggests a sizable proportion of the sample were experiencing key symptoms including counter-factual thinking, rumination, worry, and flashbacks^53^. The comparatively high rates of probable AD diagnoses in our sample underscore the challenges faced by Hong Kong migrants in adapting to their new life in the UK, which has translated into deteriorating mental well-being. The prevalence of AD among this group of migrants echoes the allegations of insufficient mental health support to migrants, as discussed in the reports by Abdul^54^ and Benson et al.^55^. The unprecedentedly high mental health needs also contrast with the mental health advantage as indicated by conventional migrant health studies^2^.

If the mental well-being of Hong Kong migrants had adhered to the typical trajectory observed in existing migrant studies on ‘healthy migrant effects’, we would expect a decline in mental well-being over time following an initial advantage^5^. However, the trend analysis (Fig. 1) suggests that the passage of time did not influence the severity of AD prevalence. The prevalence of a probable AD diagnosis remains high for the first batch of Hong Kong migrants who have settled in the UK for almost two years since mid-2020, with no sign of further deterioration or improvement. Such chronicity of AD echoes the arguments proposed by Casey^56^ and O’Donnell et al.^33^ that AD should not be viewed as a transient disorder. The persistent distress can also be understood through Lazarus and Folkman’s transactional model of stress and coping^9^. Hong Kong migrants appear to perceive their displacement as a continuous challenge that taxes their coping capacity without resulting in effective adaptation, thereby sustaining a high level of psychological strain over time.

### Building resilience against chronic stressors for preventing adjustment disorder

Several stressors, including moving to a new home (41.1%), language barrier (32.9%), pressure from cultural differences (29.7%), financial problems (27.6%), and career break due to migration (24.8%) have been identified as major stressors in our sample. The BN(O) visa route was introduced after the social unrest and the emergence of the COVID-19 pandemic, and allegedly following the implementation of the NSL in Hong Kong. Considering the context of its introduction, the first batch of these migrants from Hong Kong may have only weeks to prepare for their migration, which may have potentially involved a hasty decision or inadequate preparation. Previous research also indicates that reluctant migrants are more likely to encounter financial and cultural issues post-resettlement compared to voluntary migrants^57,58^. This aligns with a key aspect of Berry’s model of acculturation^8^, where the lack of voluntary nature of migration significantly heightens the migration stress experienced and constrains the resources available for effective integration. For Hong Kong migrants who may have been reluctant to relocate to the UK, the initial appraisal of their migration journey might have been daunting and unsettling, potentially initiating a complex and prolonged coping process that persists well beyond resettlement.

However, not all stressors serve as predictors of a probable AD diagnosis. Our logistic regression analysis reveals that only certain chronic stressors, namely family conflict, conflicts at work, illness of a loved one, unemployment, too much or too little work, pressure to meet deadlines, financial problems, language barrier, pressure of cultural difference, prospect of children’s education, were associated with a potential AD diagnosis. Interestingly, no acute stressor was related to a potential AD diagnosis, contrary to findings from previous studies that highlighted acute stressors as significant predictors^51^. This accentuates the importance of further research to determine what factors may screen out for a potential AD diagnosis and whether the nature and chronicity of the stressors moderate the emergence of AD in various contexts. Considering the distinct challenges experienced by (Hong Kong) migrants that have yet to be adequately covered by the original list of stressors, the current study included additional migration-related stressors onto the stressor list. Among them ‘language barrier’, ‘pressure of cultural differences’ and ‘career break due to migration’ were few of the most endorsed items, supporting that these stressors were common and pertinent to the stress-adjustment process of our sample. While the symptom list remained consistent, rendering the calculation of AD prevalence comparable to other studies using ADNM-8, the practice of adding context-specific stressors warrants further conceptual and practical exploration. For instance, longitudinal studies with a similar sample of Hong Kong migrants may be used for examining the temporal stability of these newly added stressors and their relationships with the occurrence of AD. The newly added stressors may also be suitable for migrants in the UK with a similar background as our sample, or Hong Kong migrants who have moved to other host countries. Future studies are needed to examine the generalizability of this new set of stressors pertinent to migration-related adjustment.

Despite its cross-sectional nature, this study adds to the existing literature on migrants’ mental health by revealing the evolution of stressors over time. The linear regression results (Fig. 2) indicate that the prevalence of some stressors such as unemployment and career break due to migration decreases statistically over time, whereas the prevalence of other stressors such as the death of a loved one, serious accident, and illness of a loved one significantly increases with time. These findings portray a picture that this group of migrants gradually reintegrates into the labour market during their first two years of relocation, leading to a notable decrease in work-related stress. However, as many of them have family members remaining in Hong Kong, post-migration difficulties stemming from concerns about family members intensified over time. Similar trends are observed in resettled reluctant migrants elsewhere^59^. While unemployment and illness of a loved one are significant predictors of a probable AD diagnosis, the time-dependent nature of the stressors may imply AD screening should change over time. By recognising the shifting nature of chronic stressors, tailored mental health support can proactively build resilience and improve long-term wellbeing. Such longitudinal observation of AD stressors is rare, and this study offers important empirical evidence to examine the life course of a stressful life event and its relevant stressors.

### Understanding the social determinants of adjustment disorder

The likelihood of AD diagnosis also differs significantly by an individual’s education level and pre-existing mental disorder. Hong Kong migrants constitute a socially and culturally homogenous group with most sharing a similar ethnic origin from the geographically compact metropolitan^60^. Thus, we could assume this group having similar levels of literacy and being exposed to similar cultural impetus regarding mental health stigma and help-seeking. According to the conservation of resource theory^61^, adjustment to a personally salient stressful event taxes an individual’s psychological and social resources; thus, those with little to begin with may experience more detrimental resource depletion, or even a caravan of resource loss. Education level, which may index a person’s socio-economic position, as well as pre-existing mental disorder which may imply a person’s psychological resilience, may represent how much resources a person has on hand to ‘spend’ for adjusting to migration. Das-Munshi et al.^62^ described mental health disadvantages as a result of downward social mobility and hardship stemming from changes in socioeconomic status. Brydsten et al.^63^ suggested that lower education levels are associated with unemployment, precarious employment, and economic deprivation, which could be precipitating factors for psychological distress. Individuals with low levels of education may face limitations in the proficiency of the language of the host country, which restricts job opportunities and integration experience, leading to further stress in daily life^64^. Given that language proficiency and job-related events were identified as significant stressors in this study, it is reasonable to conjecture that AD during migration is moderated through the socio-economic circumstances faced by low-education individuals. The association between a probable AD diagnosis with pre-existing mental disorder also comes as no surprise as depression and anxiety symptoms are commonly seen in patients with an AD diagnosis in clinical settings^28,34^. Previous studies had noted the problems in differentiating between AD and other mental health conditions and the problem of seeing AD as a ‘residual diagnosis’^65,66^. Our findings urge further studies to verify the effect of pre-existing mental disorders on the development and prognosis of AD.

### Implication and limitation

To fully realize the target for SDG3 in a world of heightened migration trends, it’s imperative to address the mental health of migrants in visa and integration policies. To the best of our knowledge, the current study is the first time AD is applied to a population facing distress from migration adopting the ICD-11 diagnostic criteria. It is also one of the very few studies that explore the social determinants of AD and the development of AD across time in the context of migration. Meanwhile, the data collected from this study also provides a close look at the mental health of Hong Kong migrants since the launch of the bespoke ‘safe and legal’ BN(O) visa route in 2021, a policy due to be reviewed in 2026.

The survey data affords us insights into the challenges confronted by the Hong Kong community upon their arrival in the UK. The decade-long cohort study with local citizens in Hong Kong conducted by Ni et al.^67^ revealed a staggering surge in mental health burdens amidst the social unrest that engulfed the city in 2019-20. Strikingly, 20% of adults reported either probable depression or suspected PTSD during the social unrest. A recent community survey spearheaded by Liang^40^, targeting Hong Kong migrants in the UK, underscored the profound and enduring impact of the social unrest and implementation of HK’s NSL on the mental well-being of the Hong Kong diaspora. From a policy perspective, our results raise concerns regarding the adequacy of mental health support in the UK, as indicated by findings that (1) an exceptionally high proportion of Hong Kong migrants are experiencing a probable AD diagnosis, (2) a probable AD diagnosis could persist among Hong Kong migrants after two years of relocation, potentially introducing a chronic illness trajectory, (3) financial and cultural stressors undermine the post-resettlement mental well-being among this group of migrants, and (4) chronic stressors would facilitate the diagnosis of AD in post-migration setting. While the bespoke BN(O) visa route for Hong Kong citizens is often portrayed by the UK government as a commitment to the ‘safe and legal’ protection of the most vulnerable, our study underscores significant reminders of how the weight of a humanitarian crisis could have extended beyond the native soil, burdening individuals during their resettlement in a new ‘home’. Moving forward, public policies should acknowledge migration as a determinant of mental well-being and recognize that both the migration process and associated stressors influence health outcomes and impede progress toward achieving SDG3. Assessment by demographics and migration-related stressors as risk factors for AD could be conducted at migrant-targeted services and community support. Migrant-targeted mental health support should also be multifaceted, targeting work, school and cultural integration, as well as family communication across borders, beyond conventional psychotherapies. This study provided insights of how factors such as employment opportunity and language skills could serve as protective factors against mental health deterioration, equipping migrants with the social and cultural resources necessary to build stability and resilience in a new environment.

Conventional studies on migrant health primarily focus on voluntary migration in Western countries, attributing the observed health advantage to the ‘selection’ effect in the migration process. Our study challenges the applicability of this health advantage in the context of reluctant migration. We reckon a proportion of Hong Kong migrants to the UK via the BN(O) visa is ‘reluctant’ as the scheme is a humanitarian visa route by default, and the ‘selection’ involved in the migration process may therefore differ fundamentally from fully voluntary migration. We highlight the discrepancy between our findings and the conventional migrant health studies that are often based on non-humanitarian visa routes, while also emphasizing the need for caution in interpreting our results. Future research should examine how resilience develops over time in response to chronic stressors, particularly in the context of migration. Understanding the dynamic interplay between persistent stressors and protective factors may inform intervention strategies that promote psychological adaptation and mitigate the long-term risks associated with migration-related distress.

To the best of our knowledge there’s no targeted interventions currently exist specifically for addressing AD among migrants in the UK. However, there have been broader efforts to improve access to mental health support, including community-based initiatives and the removal of systemic barriers. For instance in 2024, a dedicated £310,000 grant scheme was allocated to support mental health and wellbeing initiatives for BN(O) visa holders and their families across the UK^68^. Psychological interventions for AD generally focus on enhancing personal and social coping resources, with cognitive behavioural therapy (CBT) being particularly effective in alleviating AD-related distress^69^. Evidence supports the utility of bibliotherapeutic self-help manuals^70^, which could be tailored with practical guidance for migrant populations. E-health has also been proposed as a promising delivery model^71^, and recent findings indicate that technology-supported CBT interventions - including virtual and internet-based formats - show superior outcomes compared to control groups^72^. Therefore, we recommend a CBT-based approach delivered through a blended model to complement and enhance existing mental health services for migrants potentially experiencing AD.

A few limitations to our study deserve mention. First, despite garnering a substantial number of responses, the convenience sampling method employed in this study may introduce selection bias towards specific demographic subgroups, rendering the findings non-representative of the broader population. We also acknowledge the probability that convenience sampling may also inflate disorder prevalence and as such readers should interpret the results with caution. Future research should strive to replicate our study employing random sampling techniques to ensure more accurate insights. Second, mental health assessments reliant on self-reported health status often led to underreporting of stress levels^73^ among Asians due to cultural norms. The absence of diagnoses in a clinical setting in our study may potentially underestimate the true prevalence of AD. Although the scoring of ADNM-8 relied on the symptom list and impairment criterion, which were unrevised in the current study and making our scores and prevalence comparable to other studies using the scale, we call for future studies to validate our revised stressor list for migrants, considering the importance of AD in migrant mental health based our findings. In addition, even though we asked participants to indicate the presence of pre-existing mental disorders, we did not ask for detailed information such as diagnoses and time of onset in the hope that this would reduce their reluctance to report and yield more accurate information about the prevalence of pre-existing disorders. Yet, this arrangement prohibited more nuanced exploration. Future studies may delve into how pre-existing mental disorders impact AD trajectories with more nuanced surveys. Third, our research design adopted a cross-sectional approach rather than a longitudinal one. Consequently, there is limited evidence to conclusively establish a two-year duration for AD within individual cases. Additionally, some stressors such as changes in employment and circumstances of families in the place of origin, as well as the impacts of the evolving socio-political situations in the place of origin on migrants’ mental health may take more time to reveal. Thus, with most participants having arrived in the UK for only 1 to 2 years, there may not be a long-enough time frame for revealing how duration of stay affects adjustment. Longitudinal studies tracking AD progression within the same cohort are imperative to elucidate the temporal evolution of AD. Fourth, as highlighted by O’Donnell et al.^35^, discerning whether the stressor itself (e.g., financial problems) or its consequences (e.g., the inability to meet daily needs) serve as the primary trigger remains challenging. Also, whether the stressors are acute or chronic could also be an arbitrary differentiation. For instance, an accidence could be an acute stressor, while the disability it caused could be chronic. While the low incidence of acute stressors may have been responsible for a null effect in the current sample, future studies may elaborate how different types of stressors differentially affect migrants’ mental health and moderated by accessibility to services and social capital in greater nuances. Fifth, we have no variables to measure the level of reluctance or voluntariness of migration as perceived by the participants. Our assumption about the potential reluctance among a portion of the BN(O) migrant population based on the humanitarian nature of the visa route, which, according to Berry’s acculturation model^8^, is a crucial factor in governing the acculturative stress level and coping resource. However, in the absence of direct measures, our capacity to examine the nuanced effects of migration on the development of AD remains constrained. Other information deemed sensitive at the time of data collection but pertinent in migration studies, such as income and premigration trauma, were not included, reducing the comprehensiveness of the exploration. Finally, we have not included protective psychosocial factors, such as social support, language proficiency, in the current study. While our study has highlighted that younger migrants were more vulnerable, Verelst et al.^74^ found that family support serves as a protective factor against the impact of discrimination, whereas friends support reduces PTSD in young newcomers. English proficiency, a vital psychological and social resource in the UK migration context and a common mediator in Berry’s acculturation model^75^, was not directly assessed in our study, aside from its inclusion as one of the stressors within the AD model. Future studies may explore how different types of social capital differentially impact migrants’ adjustment and incorporate protective psychosocial factor to more robustly test causal claims and provide a deeper understanding of how initial resources and migration volition moderate the development of AD among migrants.

## Conclusion

Drawing evidence from an online survey conducted in the UK targeting Hong Kong migrants arriving after mid-2020, we found that the prevalence of AD among Hong Kong migrants is up to 31.8%. Unlike other stressful events, which typically cause AD symptoms to last less than six months after they end, resettlement in a new country could result in a high prevalence of AD persisting for at least two years post-arrival. Our results also reveal that chronic stressors are statistically more significant in predicting AD in the context of migration, providing crucial empirical evidence for the life course of AD with stressful life events. This study further finds that Hong Kong migrants who are younger, with lower education backgrounds and pre-existing mental disorders are more prone to AD.

Migration is an exceptionally complex process, and there’s still much to be examined regarding its psychological adjustment. Our study, using Hong Kong migrants as a case study, illustrates how individuals leaving their homeland due to social unrest may encounter significant stress and disorder during resettlement in a new country. The study signifies the importance of policy support and highlights potential policy gaps for migrants arriving through humanitarian visas. Further research is warranted to explore the life course of stressors and AD to address health inequities among migrants, especially considering projections indicating a further intensification of involuntary migration.

## Supplementary Information

Below is the link to the electronic supplementary material.

Supplementary Material 1

## Data Availability

The datasets generated and/or analysed during the current study are not publicly available due to privacy concerns associated with participant information and the proprietary nature of the data. The accessible link to the survey is no longer available, having expired following the completion of data collection. However, data are available from the corresponding author on reasonable request.
