# Supplementary material for: The pains amidst starting a new life: investigating adjustment disorder in Hong Kong migrants’ transition to the UK
Source: Sci Rep. 2025 Nov 18;15:40545. doi: 10.1038/s41598-025-24311-1 (PMC12627445; doi:10.1038/s41598-025-24311-1)
Supplement: Supplementary file 1 — Supplementary Material 1 [file 41598_2025_24311_MOESM1_ESM.docx]

Supplementary information

Table S1. Description of the base level for variables used in Table 2

| **Code for variables used in Table 2** | | | | | |
| --- | --- | --- | --- | --- | --- |
| **Age** | **Code** |  | **Education** | **Code** |  |
| 18-24 | 1 |  | Junior secondary or lower | 1 |  |
| 25-29 | 2 |  | Higher secondary education | 2 |  |
| 30-34 | 3 |  | Undergraduate | 3 |  |
| 34-39 | 4 |  | Master or above | 4 |  |
| 40-44 | 5 |  |  |  |  |
| 45-49 | 6 |  | **Family with children** |  |  |
| 50-54 | 7 |  | Yes | 1 |  |
| 55-59 | 8 |  | No | 2 |  |
|  |  |  |  |  |  |
| **Gender** |  |  | **Migrating with family member** |  |  |
| Male | 1 |  | Yes | 1 |  |
| Female | 2 |  | No | 2 |  |
|  |  |  |  |  |  |
| **Chronic illness record** |  |  | **Mental illness record** |  |  |
| Yes | 1 |  | Yes | 1 |  |
| No | 2 |  | No | 2 |  |
|  |  |  |  |  |  |
| **Previous UK living experience** | **Code** |  | **Perceived Level of Integration** | **Code** |  |
| Yes | 1 |  | Very poor | 1 |  |
| No | 2 |  | Fair | 2 |  |
|  |  |  | Below average | 3 |  |
| **Duration of residence** | |  | Average | 4 |  |
| 0-6 months | 1 |  | Above average | 5 |  |
| 6-12 months | 2 |  | Good | 6 |  |
| 12-18 months | 3 |  | Very good | 7 |  |
| 18 months or more | 4 |  |  |  |  |
|  |  |  |  |  |  |

Table S2. Questionnaire used for this study

Screening question

Here we would like to learn about your current migration state. Please choose from below your migration status.

I am currently residing in the UK/ I am currently residing outside Hong Kong, but not in the UK/ I still reside in Hong Kong but I have applied for the BN(O) visa to come to the UK

I still reside in Hong Kong and I am considering applying for the BN(O) visa to come to the UK/ I still reside in Hong Kong and I will leave Hong Kong with a visa route other than BN(O) visa/ I still reside in Hong Kong and have no intention to leave Hong Kong

Part 1 - Basic background about migration

1. By what visa route did you choose to come to the UK?

BN(O) visa route (main applicant)/ BN(O) visa route (dependent)/ working visa or global talent or student visa/ BC citizen or spouse/Asylum or refugee/Others

2. How long have you moved to the UK?

Within 6 months/ 6-12 months /12-18 months/ Over 18 months

3. Do you have previous experience living in the UK?

Yes/No

4. If yes, how long?

(Fill in)

Part II – Acculturation

1. In general, how well do you find yourself settling down in the UK since your arrival?

Very poor/Fair/Below average/Average/Above average/Good/Very good

Part III - Adjustment disorder

1. Below is a list of stressful events relating to migration to the UK. Please indicate those events that happened after you relocated to the UK and are currently a strong burden to you, or have burdened you in the last six months. You can indicate as many events as applicable.

Divorce or separation/Family conflicts/conflicts at work/Conflicts with neighbors/Illness of a loved one/Death of a loved one/Adjustment due to retirement/Unemployment/Too much or too little work/Pressure to meet deadlines or time pressure/ Moving to a new home/Language/ Financial problems/ Own serious illness/ Risk of criminal prosecution/Racial discrimination/Serious accident/Pressure of cultural difference/Career break due to migration/Prospect of children in education/Any other stressful event (please indicate)

In the following, you will find various statements about which reactions these types of stressful events can trigger. We would like to ask whether you have been having these reactions for the past 6 months

2. I have to think about the stressful events repeatedly

Never/rarely/sometimes/often

3. I have to think about the stressful events a lot and this is a great burden for me

Never/rarely/sometimes/often

4. I constantly get memories of the stressful events and can’t do anything to stop them

Never/rarely/sometimes/often

5. My thoughts often revolve around anything related to the stressful events

Never/rarely/sometimes/often

6. Since the stressful events, I find it difficult to concentrate on certain things

Never/rarely/sometimes/often

7. Since the stressful events, I do not like going to work or carrying out the necessary tasks in everyday life

Never/rarely/sometimes/often

8. Since the stressful events, I can no longer sleep properly

Never/rarely/sometimes/often

9. All in all, the situation causes serious impairment in my social or occupational life, my leisure time, and other important areas of functioning.

Never/rarely/sometimes/often

**Part IV - Demographic Information**

1. What is your age group?

18 to 24/25 to 29/30 to 34/35 to 39/40 to 44/45 to 49/50 to 54/55 to 59/60 or above/ Prefer not to say

2. What is your gender?

Male/Female/Prefer not to say

3. What is your highest education level? (Before graduation is also counted)

Junior Secondary (e.g. HKCEE, GCSE) or below/Higher Secondary (e.g. HKDSE/HK A-level/A-level)/Undergraduate level or postsecondary/Master or above/

Don’t know/Prefer not to say

4. Do you have children?

Yes/No/Prefer not to say

5. How many family members come to the UK with you?

0/1/2/3/4/5/6 or more /Prefer not to say

6. Do you need help with emotional or mental health problems, such as feeling sad, anxious, or nervous during the 12 months?

Yes/No/ Prefer not to say

7. Do you have any chronic illness records?

Yes/No/ Prefer not to say


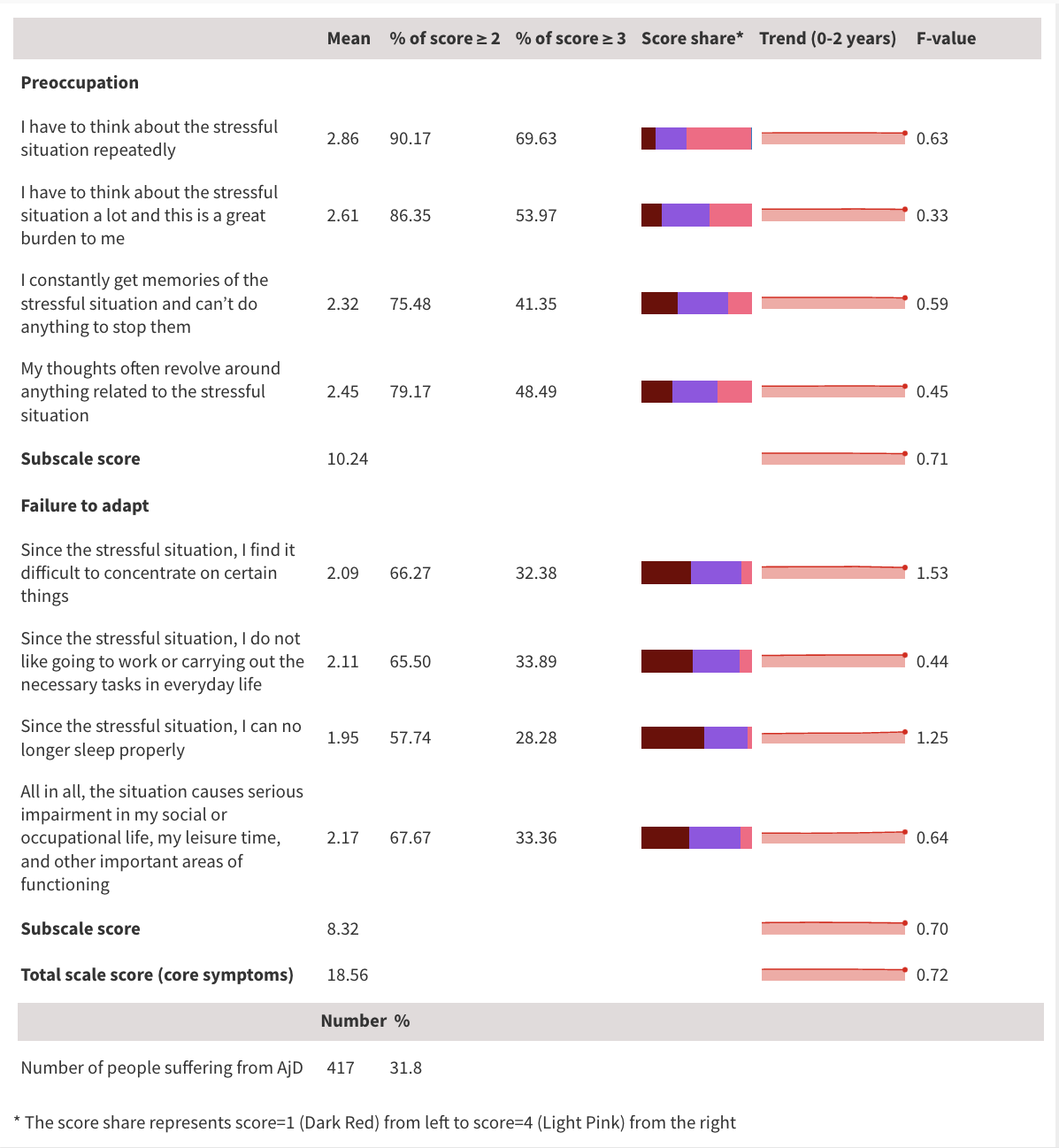


Figure S1. Breakdown of results of ADNM-8 for the Hong Kong migrants. The score share is visualized under the column ‘Score share’. The life course of symptoms during the first two years of migration is visualized in pink.
